# A First Report on Experience in Managing Infants with Congenital Toxoplasmosis in Ethiopia: Case Reports and a Review of Evaluation and Treatment

**DOI:** 10.1155/2021/9934391

**Published:** 2021-04-15

**Authors:** Tinsae Alemayehu, Selamawit Assefa, Solomie Jebessa Deribessa, Semienew Ambachew

**Affiliations:** ^1^American Medical Center, Addis Ababa, Ethiopia; ^2^Division of Pediatric Infectious Diseases, Department of Pediatrics and Child Health, St. Paul's Hospital Millennium Medical College, Addis Ababa, Ethiopia; ^3^Lancet Specialized Internal Medicine and Surgical Center, Addis Ababa, Ethiopia; ^4^Division of Neonatology, Department of Pediatrics and Child Health, Addis Ababa University, Addis Ababa, Ethiopia

## Abstract

*Background*. Congenital toxoplasmosis is a major sequela of untreated primary maternal infection. With or without symptoms, untreated infections eventually lead to multiple neurologic complications. Despite the high *Toxoplasma gondii* seroprevalence in the Ethiopian population, there are no reports of newborns diagnosed and treated for congenital toxoplasmosis. *Presentation of Cases*. The clinical presentation, evaluation, and management of three infants with congenital toxoplasmosis are described. Two were symptomatic at birth. All three had confirmed diagnoses using *Toxoplasma* serologic tests. Two completed their treatment with one infant developing complications of strabismus and seizure disorder. *Discussion and Conclusions*. There is little experience in managing congenital toxoplasmosis in Ethiopia due to constraints in diagnostics and therapy. The description of this first such report underscores the need for risk assessment and evaluation during antenatal care to obtain favorable fetal outcomes.

## 1. Background

Congenital toxoplasmosis (CT) is a major sequela of untreated primary maternal infection [1]. Though the risk for mother-to-fetus transmission is highest in the 3^rd^ trimester of pregnancy, early pregnancy transmission carries the highest likelihood for complicated CT [2]. Immune-compromised mothers with reactivation of bradyzoites during pregnancy and mothers who had acquired the infection within the three months preceding conception have additional risks for their newborns to acquire CT [3]. A mother acquires primary toxoplasmosis via ingestion of undercooked meat containing tissue cysts or water, fruits and vegetables with cat feces, or soil harboring the oocysts which can survive for more than a year in suitable environments. Insects such as flies and cockroaches can also transport oocysts to food items [4].

While most infants are asymptomatic in their neonatal period, untreated almost all develop ocular and/or neurologic sequelae after many months to years [5]. Classic signs for symptomatic CT are chorioretinitis, hydrocephalus, and brain parenchymal calcifications. But symptomatic infants may be born premature with intrauterine growth retardation, low birth weight, jaundice, petechiae, hepatosplenomegaly, myocarditis, diarrhea, hearing loss, nephrotic syndrome, anemia including erythroblastosis fetalis, maculopapular rash, and pneumonia [6]. Diagnosis is made via serologic or PCR tests of blood, CSF, urine, etc. Timely treatment improves intellectual function, leads to regressions of retinal lesions, prevents hearing loss, and reduces the risk of seizures [3].

Though many studies have reported that three-fourth of pregnant mothers in antenatal clinics in Ethiopia are positive for anti-*Toxoplasma* IgG [7–11], there are no descriptions of clinical experiences involving newborns with CT in Ethiopia. The objective of this case report is to share its clinical findings and inform our local practice bereft of experience in similar syndromes.

## 2. Description of Cases

### 2.1. Case 1

A 12-day-old female newborn was referred to our clinic after her mother was diagnosed with neurotoxoplasmosis during the 3^rd^ trimester of pregnancy. The newborn was delivered at term to her 39-year-old para IV mother who had been diagnosed with HIV infection a year ago but opted out of treatment. During pregnancy, she had a single antenatal care follow-up visit. At 34 weeks of gestation, the mother presented with lower extremity weakness, drooping of the left eye, and diplopia of 5-day duration. She reported no dietary risks like raw meat and dairy consumption and contact with cats in her household. Her physical examination showed normal vital signs, left-sided blurry disc on fundoscopy examination, anisocoria, left-sided ptosis with impaired upward gaze, nystagmus, and decreased power of left side of her body (arms 4/5 and legs 3/5).

Her lab workup showed hemoglobin of 10 g/dl (normal for age 12.5–16 g/dl), mean corpuscular volume (MCV) of 77 fl (78–100 fl), white blood cells of 670/mm^3^ (4000–10500/mm^3^), platelets of 125,000/mm^3^ (normal 150000–400000/mm^3^), and CD4 of 127/mm^3^ (normal 500–1200/mm^3^) (her HIV viral load could not be determined) and brain CT showing multiple ring-enhancing lesions over the basal ganglia suggestive of neurotoxoplasmosis. She started treatment with trimethoprim-sulfamethoxazole, antiretroviral treatment with tenofovir disoproxil fumarate (TDF), lamivudine (3TC), and efavirenz (EFV), and intravenous dexamethasone.

The newborn was asymptomatic at birth and had no abnormal signs with a birth weight of 4600 grams and head circumference of 34.5 cm. Her complete blood count (CBC), serum bilirubin, and brain ultrasound were all normal. She had elevated *Toxoplasma* IgM of 0.96 IU/mL (normal <0.5 IU/mL) and *Toxoplasma* IgG of 9.1 IU/mL (normal <3 IU/mL). Details of the commercial test kit could not be extracted. Nevirapine prophylaxis was initiated at birth and the mother opted for exclusive breastfeeding. After a few visits, the newborn was lost from follow-up despite appointments at the pediatric ophthalmology and infectious diseases' clinics and parents being given prescriptions for pyrimethamine, sulfadiazine, and leucovorin. Reestablishment of contact by phone communication was not successful.

### 2.2. Case 2

A 3-week-old male infant presented with persistent yellowish discoloration of skin since birth. He was born at a gestational age of 34 weeks and 3 days. His mother's antenatal period was complicated by Rh hemolytic disease-induced hydrops fetalis for which she received four rounds of in utero transfusions. He was delivered via C-section with a birth weight of 2400 grams. The newborn received an exchange transfusion for neonatal hyperbilirubinemia due to Rh hemolytic disease in addition to intensive phototherapy. Persistence of the anemia, thrombocytopenia, and direct hyperbilirubinemia during his stay at the neonatal intensive care unit prompted a workup for congenital infections. His abdominal ultrasound showed normal findings. His serum VDRL was negative. He had negative CMV IgM and elevated CMV IgG (60.9 IU/mL: normal <6 IU/mL). He had positive *Toxoplasma* IgM and elevated *Toxoplasma* IgG of 9.4 IU/mL (normal <3 IU/mL). Details of the commercial test kit could not be extracted. His CSF analysis showed 2 cells/mm^3^ lymphocytes and protein of 151 mg/dL (upper limit of normal for his age being 115 mg/dL).

**Figure 1:**
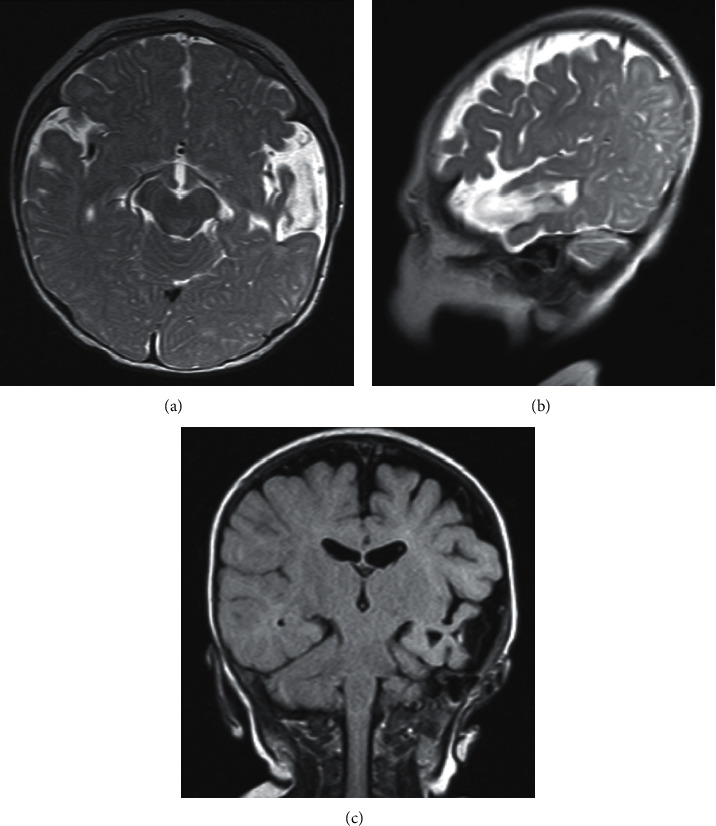
(a)-(c) Brain MRI (T2, T1, and T2 flair images) of the second patient showing left temporal lobe peripheral and periventricular focal encephalomalacia.

He was started on oral pyrimethamine, sulfadiazine, and leucovorin to be continued till 1 year of age. There was no chorioretinitis on ophthalmologic evaluations at 6 and 14 weeks of postnatal age. He had a normal transfontanelle ultrasound (MRI testing was unavailable in his early infancy). The infant's anthropometric progression was within normal ranges: his weight, length, and head circumference fluctuating between 3^rd^ and 50^th^ centiles for age through the course of his first year. MRI of the brain at 8 months of age showed left temporal lobe peripheral and periventricular focal encephalomalacia: possibly an old ischemic insult with no midline shift (Figures 1(a)–1(c)).

Audiogram examination at 1 year of age showed normal hearing ranges. No adverse reactions for therapeutic drugs were seen during clinical and complete blood count (CBC) evaluations in the follow-up period. The infant's developmental milestones were comparable to his peers at 1 year of age. Concern for the development of seizures remains because of his left temporal lobe focal encephalomalacia and he is undergoing clinical and ophthalmologic follow-up evaluations (planned every two months for the second year of life, every three months for the third year of life, and every three to six months afterwards) [3].

### 2.3. Case 3

A 4-year-old boy was referred for the continuation of follow-up for CT after completing his anti-*Toxoplasma* treatment in his first year of life. He was born at 37 weeks of gestational age after an uneventful pregnancy. His birth weight was 3000 grams. After presenting with increased head size with a head circumference of 42 cm (between +2 and + 3 SD for his age) and abnormal body movements at 2 months of age, he underwent an MRI of the brain which showed communicating hydrocephalus. Subsequent workup showed positive *Toxoplasma* IgM and elevated *Toxoplasma* IgG of 21 IU/mL (normal <3 IU/mL). Details of the commercial test kit could not be extracted. He was initiated on oral levetiracetam and underwent ventriculostomy. An ophthalmologic assessment showed bilateral strabismus (esotropia). A lumbar puncture was deferred. He was treated with oral pyrimethamine, sulfadiazine, and leucovorin for 1 year. He started wearing corrective eyeglasses at 6 months of age. With control of seizures, levetiracetam was discontinued at one year of age but was restarted after the reappearance of generalized tonic seizures at 3 years and 5 months of age. He achieved his developmental milestones in comparable time to his peers. An audiogram done at 3 years of age showed normal hearing ranges.

At 4 years of age (start of follow-up at our clinic), he was asymptomatic and had good seizure control. His anthropometric measurements showed a height of 111 cm (50^th^–75^th^ centile for age), a weight of 17 kg (25^th^–50^th^ centile), and a head circumference of 52 cm (+1 SD). He remained wearing glasses for his bilateral strabismus.

## 3. Discussion

Fifteen million people are living with CT with an estimated global incidence of CT of 190,100 cases per year and an incidence of 1.5 cases per 1000 live births [12]. Primary toxoplasmosis during pregnancy carries many adverse effects on the fetus. A study from Senegal shows women who miscarried had a two times risk of *Toxoplasma* seroconversion compared to those who had not [13]. Epidemiologic studies of CT in Africa are represented by case reports from a few countries, appearing in the literature as early as the 1950s [14]. Only a single study from the continent identifies genotype distribution in acute *Toxoplasma* infections during pregnancy, with *Toxoplasma* genotype 2 dominating among a Tunisian cohort [15].

Toxoplasmosis is likely to be transmitted from mother to fetus by placental invasion by tachyzoites leading to their multiplication within placental cells and some tachyzoites accessing fetal circulation or transmitted during vaginal delivery [16]. Maternal immune-compromise (as in our first case) and primary infection during pregnancy and within three months before conception confer a higher risk of mother-to-fetus transmission [3]. Antenatal consumption of raw meat carries varying degrees of risk for transmission, depending on the type of animal meat consumption [17]. Studies in Ethiopia indicate a high seroprevalence of *Toxoplasma* IgG among food and domestic animals of 34.5% (sheep and goats), cattle (10.7%), camels (14.4%), and cats (88%) [18, 19]. Oocyst shedding among cats was also noted to be 17.5% according to one systematic review [19]. All mothers in our case reports did not recall consuming raw meat during their pregnancies, but two reported the presence of pet cats in their households.

The majority of newborns affected by CT are asymptomatic. In most cohorts around the world, the prevalence of even a single symptom being present among newborns with CT is 20–27% [6, 20]. In our series, two of three patients were symptomatic. Such discrepancies may be because infectious diseases' consultations are made for symptomatic cases and because asymptomatic ones are not brought to medical attention. If untreated, almost all affected children will be symptomatic as adolescents. Prematurity and intrauterine growth retardation may be seen in affected infants (as seen in one of our cases), especially when transmission occurs before 20 weeks of gestation [21] as seen in one of our cases. The commonest ocular complications are chorioretinitis, strabismus, microphthalmia, cataracts, glaucoma, nystagmus, optic neuritis, etc., and these may relapse during late childhood. Less common complications include seizures, hydrocephalus, and brain atrophy [22]. Upon latest evaluations, one of our cases had complicated with strabismus and seizure disorder.

Radiologic findings of CT include cerebral or cerebellar atrophy, encephalomalacia, cortical or subcortical calcifications, ventriculomegaly, and micro- or macrocephaly [23]. Our third patient had communicating hydrocephalus while our second patient had unilateral encephalomalacia (Figures 1(a)–1(c)). Laboratory abnormalities include persistent anemia and thrombocytopenia as seen in our second patient, direct hyperbilirubinemia as seen in our second patient, eosinophilia (infrequent), and abnormal CSF parameters (in half of the affected newborns) of elevated CSF protein (beyond 1000 mg/dl in severe cases) [6]. One patient had a mildly elevated CSF protein for his age. Though a lumbar puncture was deferred in another patient who had hydrocephalus, CSF protein of above 1000 mg/dL frequently accompanies hydrocephalus in newborns with CT [24], and hence, elevated CSF protein may likely present.

Etiologic diagnosis in CT is settled by either serologic tests or PCR of body fluids. A diagnosis of CT can be made if a newborn has elevated *Toxoplasma*-specific IgG and positive qualitative (or elevated quantitative titers) IgM or IgA (seen in all of our patients). It can also be made if there is elevated *Toxoplasma*-specific IgG (with negative IgM and IgA) but with untreated primary infection during pregnancy. Additional ways of diagnosing CT are in cases of elevated *Toxoplasma*-specific IgG (with negative IgM and IgA) but with major neonatal clinical features (ophthalmologic, auditory, imaging findings, or a markedly elevated CSF protein with/without positive CSF *Toxoplasma* PCR) or if the newborn has a positive *Toxoplasma*-specific IgG (with negative IgM and IgA) which is increasing in titers throughout infancy or remains positive beyond one year of age (rules out the passive transfer of maternal antibodies and confirms a true neonatal infection) [25].

All CT-affected infants should receive treatment. The preferred treatment of CT consists of oral pyrimethamine, sulfadiazine, and leucovorin (to prevent bone marrow suppression) for one year [26]—a treatment course completed by two of our three patients while the other was lost to follow-up. Potential adverse effects to monitor are rash, vomiting, and diarrhea (all three drugs), reversible neutropenia and anemia (pyrimethamine and sulfadiazine), seizures (pyrimethamine), hematuria and rash (sulfadiazine), and stomatitis (leucovorin) [24]. Steroids should be added after three days of anti-*Toxoplasma* treatment if there is severe chorioretinitis, focal brain parenchymal lesions with mass effects, or CSF protein above 1000 mg/dL [3, 26]. Steroids may be given for many months till the disappearance of protein in CSF or till chorioretinitis resolves [27].

Supportive care should be given when indicated, i.e., anticonvulsants, ventriculoperitoneal shunts (contributes to favorable outcomes when performed promptly), and vitrectomy [24]. The anticonvulsant of choice is levetiracetam (as used by our third patient) because it has no significant drug-drug interactions with pyrimethamine and sulfadiazine like commonly used anticonvulsants (phenobarbital, phenytoin, and carbamazepine) [24]. Infants with CT should undergo clinical and ophthalmologic evaluations every month for the first year, every 2 months for the second year, every 3 months for the third year, and every 3–6 months afterwards [3, 6].

Poor prognostic factors in CT include delayed medical management and drainage of hydrocephalus, recurrent ventriculoperitoneal shunt infections, and severe visual impairment [28]. Consuming raw meat and contact with household pets and food animals in areas where there is a high seroprevalence of *Toxoplasma* infection among animals should be avoided by pregnant women [29]. Frequent antenatal care accompanied by imaging and serologic tests can increase the identification of acute infections during pregnancy and prevent fetal sequelae [30]. Treatment with spiramycin prevents mother-to-fetus transmission of toxoplasmosis in more than half of cases when the diagnosis is made before 18 weeks of pregnancy. Beyond 18 weeks of pregnancy, triple therapy with pyrimethamine, sulfadiazine, and leucovorin can be provided for the mother [31].

## 4. Conclusions

There have so far been no reports of congenital toxoplasmosis in Ethiopia despite a high population seroprevalence for acquired toxoplasmosis. Limited diagnostics and treatment options have led to little experience in managing this congenital infection. Our case reports describe the presentations of three Ethiopian infants with congenital toxoplasmosis and outline principles of evaluation, treatment, and follow-up for affected infants. In doing so, the description of their illnesses stimulates awareness of this diagnosis in at-risk pregnancies and underlines the need for early diagnosis and treatment for favorable fetal outcomes.

## Data Availability

All relevant data have been included within the manuscript.
